# Coronin Is a Component of the Endocytic Collar of Hyphae of *Neurospora crassa* and Is Necessary for Normal Growth and Morphogenesis

**DOI:** 10.1371/journal.pone.0038237

**Published:** 2012-05-31

**Authors:** Ramon O. Echauri-Espinosa, Olga A. Callejas-Negrete, Robert W. Roberson, Salomon Bartnicki-García, Rosa R. Mouriño-Pérez

**Affiliations:** 1 Departamento de Microbiología, Centro de Investigación Científica y de Educación Superior de Ensenada (CICESE), Ensenada, Baja California, México; 2 School of Life Sciences, Arizona State University, Tempe, Arizona, United States of America; Université de Nice-CNRS, France

## Abstract

Coronin plays a major role in the organization and dynamics of actin in yeast. To investigate the role of coronin in a filamentous fungus (*Neurospora crassa*), we examined its subcellular localization using fluorescent proteins and the phenotypic consequences of coronin gene (*crn-1*) deletion in hyphal morphogenesis, Spitzenkörper behavior and endocytosis. Coronin-GFP was localized in patches, forming a subapical collar near the hyphal apex; significantly, it was absent from the apex. The subapical patches of coronin colocalized with fimbrin, Arp2/3 complex, and actin, altogether comprising the endocytic collar. Deletion of *crn-1* resulted in reduced hyphal growth rates, distorted hyphal morphology, uneven wall thickness, and delayed establishment of polarity during germination; it also affected growth directionality and increased branching. The Spitzenkörper of Δ*crn-1* mutant was unstable; it appeared and disappeared intermittently giving rise to periods of hyphoid-like and isotropic growth respectively. Uptake of FM4-64 in Δ*crn-1* mutant indicated a partial disruption in endocytosis. These observations underscore coronin as an important component of F-actin remodeling in *N. crassa*. Although coronin is not essential in this fungus, its deletion influenced negatively the operation of the actin cytoskeleton involved in the orderly deployment of the apical growth apparatus, thus preventing normal hyphal growth and morphogenesis.

## Introduction

Over the last four decades, an extensive literature has accumulated implicating the cytoskeleton in the polarized growth of fungal hyphae but the exact role of the actin and microtubular cytoskeletons in organizing the exocytic apparatus that constructs the hyphal cell wall has yet to be elucidated. The actin cytoskeleton has been considered to be the driving force for short distance delivery of exocytic vesicles to the plasma membrane [1]–[2]. According to predictions derived from mathematical modeling and computer simulation [3], the Spitzenkörper (Spk) functions as a vesicle supply center (VSC) coordinating vesicle delivery to the plasma membrane [1], [4].

There is considerable evidence implicating both the actin and microtubular cytoskeletons in the structure and/or operation of the Spk. Evidence arises from multiple experiments including inhibitor studies e.g., [5]–[6], electron microscopy e.g., [7]–[8]; and molecular tagging with fluorescent proteins e.g., [9]–[11].

The use of filamentous actin (F-actin) disrupting chemicals (e.g., cytochalasin, latrunculin) demonstrated that actin is required for normal apical growth, maintenance of the hyphal tip shape, and polarized enzyme secretion in different filamentous fungal organisms [6], [10]–[12]. F-actin is found throughout the hyphal cytoplasm in the form of cortical actin cables lining the hyphal tube, in the core of the Spk, and in cortical patches (e.g., those forming a subapical endocytic collar behind the hyphal tips of *Aspergillus nidulans* [10], [13]–[14], *N. crassa* [11], and *Athelia (Sclerotium) rolfsii* [15].

Hyphal growth involves continuous addition of new plasma membrane, proteins, and cell wall material at the apex in a gradient fashion [1]. Theoretical calculations on the balance between membrane conveyed by the exocytoic vesicles and the new plasma membrane generated indicates the likelihood that an excess of membrane would be produced [16] (Bartnicki-Garcia, unpublished). Therefore it seems reasonable to assume that an endocytic mechanism exists that ensures that excess membrane material is efficiently reutilized, and also trans-membrane proteins recycled [16]–[21]. The spatial proximity of the exocytosis (apex) and endocytosis (subapex) sites poses the intriguing possibility that both processes may operate in tandem as part of the polarized machinery responsible for apical growth [13]–[14].

Coronin is a protein that binds to the sides of actin filaments where Arp2/3 complex activity mediates further F-actin polymerization, and thus predominantly localizes in sites of active actin remodeling [22]–[26]. Members of the coronin family contain a phosphorylation site within the N-terminal domain that regulates the interaction of coronin with other proteins, such as the Arp2/3 complex [27]–[28]; an additional characteristic is the WD40 repeat, which is known to form a β-propeller structure mediating protein-protein interactions [26], [29]–[30].

By protein sequence comparison (pBLAST), we identified the homologue of the coronin gene in the *N. crassa* genome (locus # NCU00202), and tagged it with either green fluorescent protein (GFP) or monomeric cherry fluorescent protein (mChFP). By confocal microscopy, we determined its localization and dynamics. We also examined the *crn-1* gene deletion mutant of *N. crassa* to assess phenotypic changes in polarized growth, hyphal morphology and Spk appearance and behavior.

Coronin has been found in a variety of eukaryotic organisms [31]. Ours is the first report on the localization and dynamics of coronin in a filamentous fungus. This study showed coronin located in a subapical collar of actin patches. The properties of a coronin null mutant gave us valuable insight into the role of coronin in endocytosis, hyphal growth and morphogenesis.

## Results

### CRN-1-GFP localization and colocalization with other actin binding proteins (ABPs)

**Figure 1:**
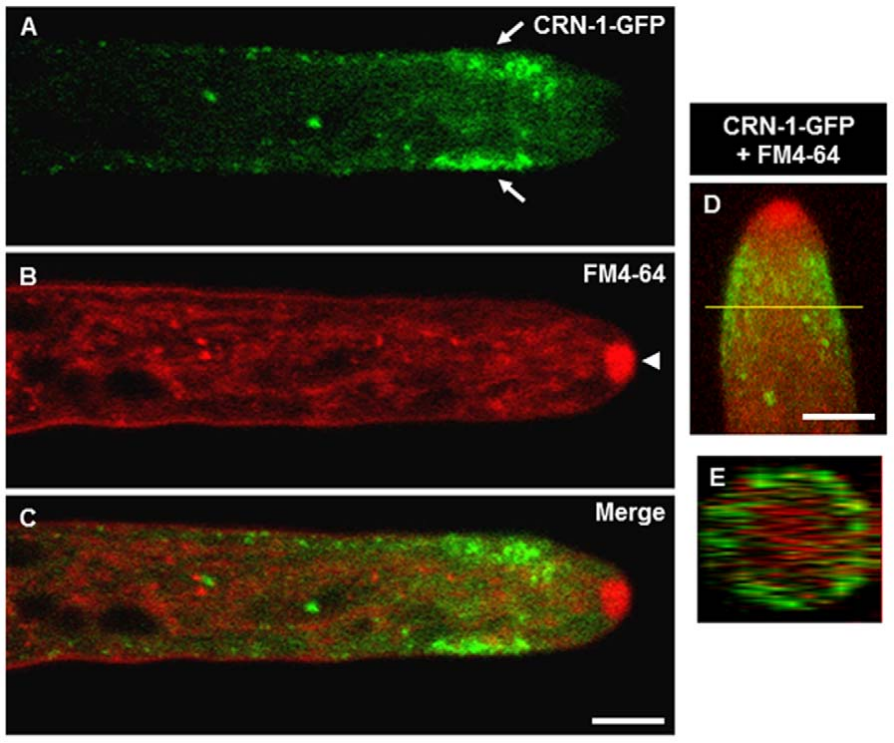
Subapical localization of coronin. (A) CRN-1-GFP forms a subapical collar along the inner perimeter of the hypha (arrows), (B) FM4-64 staining reveals the position of the Spk (arrowheads), (C) merge of CRN-1-GFP and FM4-64 staining shows the absence of CRN-1-GFP in the Spk, single confocal plane images. (D) 3D reconstruction of merged confocal z-stacks showing CRN-1-GFP and FM4-64 localization, (E) orthogonal view of the 3D reconstruction shown in (D), the yellow line indicates the position within the tip where the cross-section was taken. Scale bars = 5 µm.

CRN-1-GFP was present as small mobile cortical patches throughout the hypha, but concentrated near the hyphal apex forming a wide subapical collar (8–9 µm in width) leaving a patch-free zone of ∼4 µm in the apical region (Fig. 1A–1C). In distal parts of the hyphae, there were scattered CRN-1-GFP patches but in much lower density compared to the subapex. As the hypha elongated, the subapical collar of coronin maintained a constant distance from the hyphal tip (Supplementary Movie S1), except during occasional periods of Spk disappearance when the patches moved towards the apex (Supplementary Movie S2).

CRN-1-GFP patches appeared to localize immediately under the FM4-64-stained plasma membrane (Fig. 1C, 1E). To better visualize the architecture of the CRN-1-GFP collar, we made a 3D reconstruction of confocal z-stacks. As shown in Fig. 1D, the patches formed a nearly complete cortical ring in the hyphal subapex (Fig. 1D, 1E).

**Figure 2:**
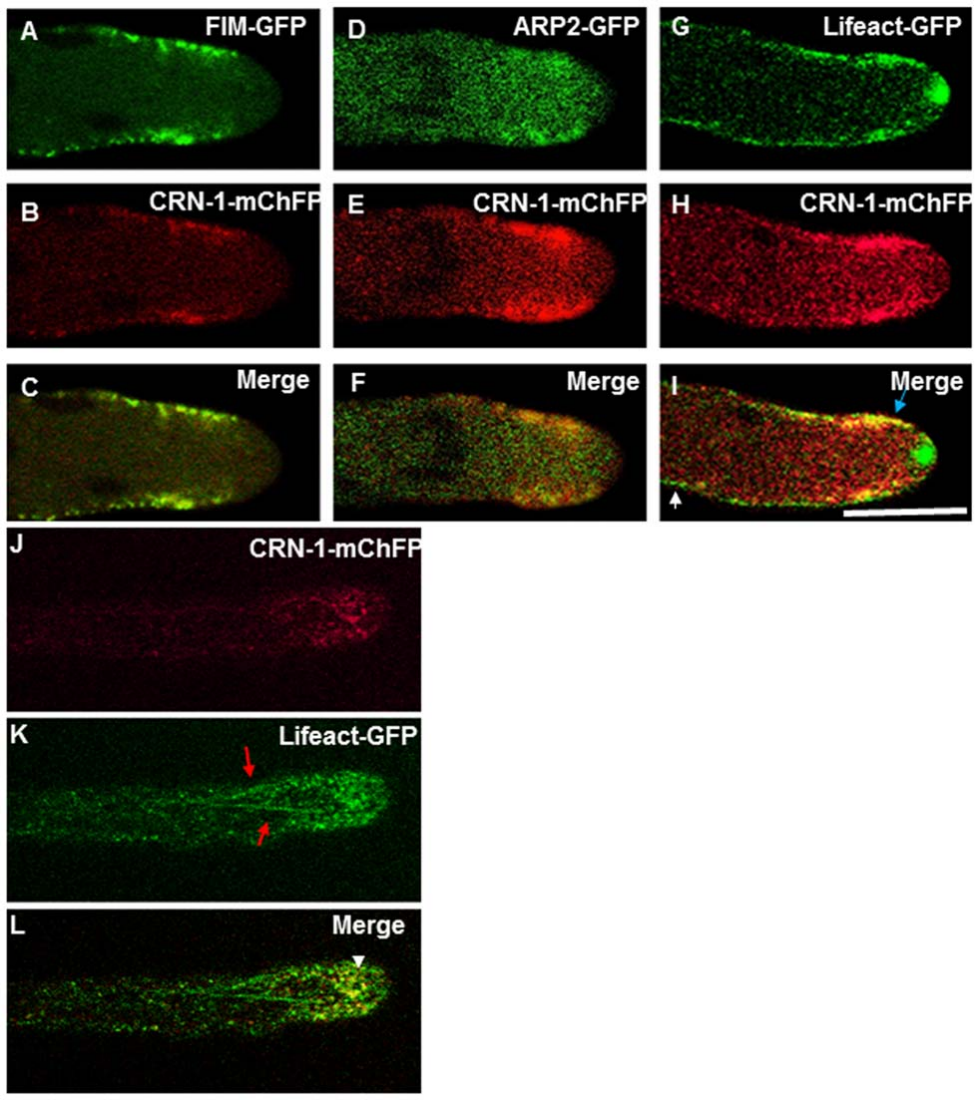
Co-expression of coronin with fimbrin, Arp2 and actin. (A–C) Colocalization of Fimbrin (FIM-GFP) and CRN-1-mChFP. (D–F) Colocalization of Arp2 (ARP-2-GFP) and CRN-1-mChFP. (G–I) Partial colocalization of the actin marker Lifeact-GFP and CRN-1-mChFP. (J–L) Co-expression of CRN-1-mChFP and Lifeact-GFP showing the lack of colocalization between coronin patches and actin cables. are depicted by. (L) Merge, not clear association of crn-1 patches is observed with actin filaments, arrowhead shows colocalization of actin patches with CRN-1-mChFP. The white arrow points a region where there is only labeling with Lifeact-GFP and the blue arrow show the patches where CRN-1-mChFP and Lifeact-GFP colocalized. Note the presence of actin in the Spk but not of patch related ABPs. The red arrows in (K) point the actin cables and the white arrowhead show the colocalization of actin and coronin in the patches subapical collar. Scale bar = 5 µm.

To examine the relationship of coronin with actin and with other ABPs during apical growth, the *N. crassa* strain expressing CRN-1-mChFP was fused vegetatively with strains expressing FIM-GFP, ARP-2-GFP or Lifeact-GFP. CRN-1-mChFP patches colocalized with fimbrin (FIM-GFP) (Fig. 2A–2C) and the Arp2/3-complex (ARP-2-GFP) (Fig. 2D–2F). Visualized with Lifeact-GFP, actin was present along the entire hyphal length examined. Some of the actin patches colocalized with the CRN-1-mChFP patches of the subapical collar (Fig. 2G–2I). A significant finding was the absence of coronin in the Spk or is immediate vicinity, as shown above, despite a strong signal for actin in the core of the Spk (Fig. 2G–2I). We did not observe coronin organized in filament arrays, which would suggest a lack of association with actin cables (Fig. 2J–2K). Instead, our data indicate that coronin associates exclusively to F-actin patches.

**Figure 3:**
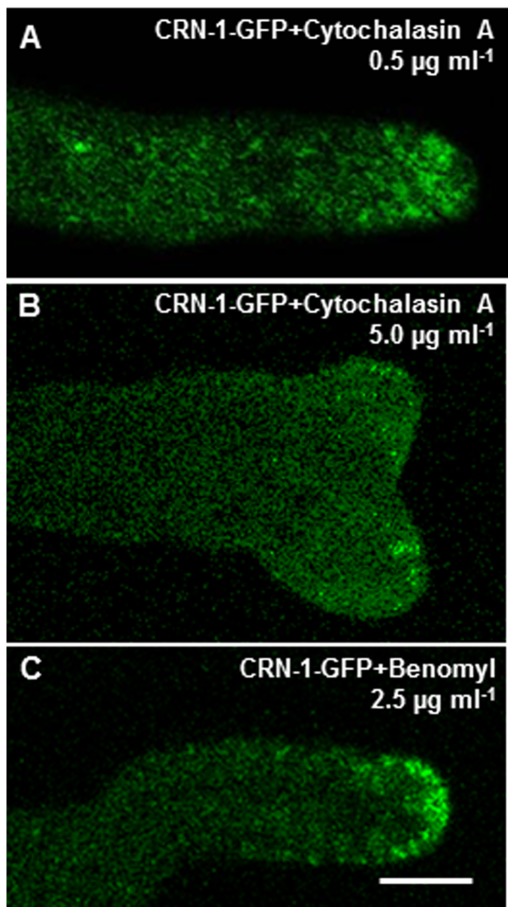
Effect of cytoskeleton depolymerization drugs on the localization and integrity of coronin patches. Hyphae exposed to: (A) the anti-actin drug, 1.0 µg ml^−1^ cytochalasin A, (B) 5.0 µg ml^−1^ cytochalasin A, and (C) the anti-tubulin drug 2.5 µg ml^−1^ benomyl. Scale bar = 5 µm.

To investigate the functional relationship between CRN-1-GFP and the main structural polymers of the cytoskeleton, we tested the effect of actin and microtubule inhibitors on CRN-1 dynamics. At a low concentration (0.5 µg ml^−1^ cytochalasin A), the collar of CRN-1-GFP patches became disorganized and the patches displaced to the apical dome (Fig. 3A). At higher concentration (5.0 µg ml^−1^), patches disappeared almost completely (Fig. 3B). On the other hand, coronin patch integrity was not affected by benomyl treatment, but the patch distribution was disrupted with the patches located in the apical dome (Fig. 3C).

### Coronin disruption phenotypes

**Figure 4:**
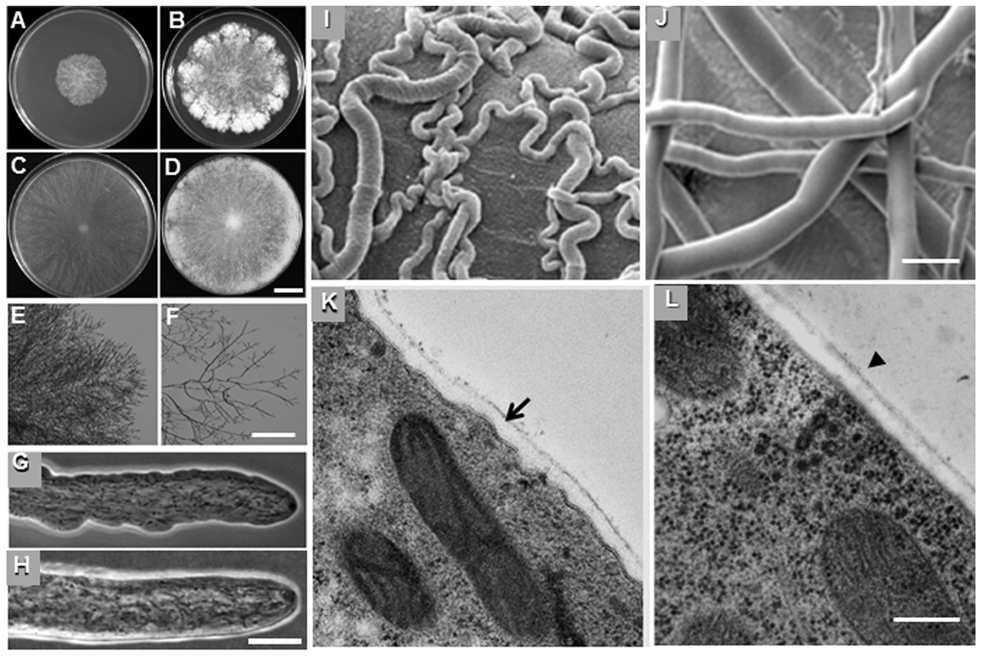
Phenotype of Δ*crn-1* mutant. **Colony morphology of (A–B) Δ** ***crn-1*** **mutant and (C–D) WT strain after 24 and 48 h of incubation on VMM at 28°C.** Low magnification images of the colony edge of (E) Δ*crn-1* mutant and (F) WT strain. Phase contrast images of hypha of (G) Δ*crn-1* mutant and (H) WT strain. SEM images of (I) the meandering phenotype of Δ*crn-1* mutant hyphae and (J) the straight WT hyphae. TEM images of the subapical region of 6 h-old germlings of (K) Δ*crn-1* mutant and (l) WT strain. A comparison of the uneven thickness of the cell wall and the ruffled plasma membrane of the mutant (arrow) with the uniform envelope of the WT (arrowhead). Scale bars = (A–D) 2.5 cm, (E–F) 100 µm, (G–J) 10 µm, (K–L) 1.0 µm.

By PCR, we corroborated the absence of *crn-1* gene in a Δ*crn-1* mat a deletion mutant provided by the Fungal Genetics Stock Center. Macroscopic and microscopic characterization of the Δ*crn-1* strain, revealed a compact slow growing, crenulated colony that conidiated poorly (Fig. 4A, 4B, Table 1). The lateral branching frequency of leading hyphae at the colony periphery was increased five-fold in the Δ*crn-1* mutant (Fig. 4E, 4F, Table 1). Hyphae of the coronin null mutant grew mostly in a meandering fashion rather than following the usual straight trajectory (Fig. 4I, 4J). The contour of the Δ*crn-1* mutant (Fig. 4G) hyphae was often irregular contrasting with the smooth outline of a WT hyphae (Fig. 4H). A telling difference was discovered by TEM showing the Δ*crn-1* mutant had an irregular hyphal cell wall of uneven thickness bordered by an undulated plasma membrane (Fig. 4K) whereas the cell wall of the WT showed the expected uniform wall thickness (Fig. 4L).

**Table 1:** Growth kinetics, conidiation rate and branching.

Strain	Elongation rate	Biomass production	Conidiation rate	Branching rate
	mm min^−1^	mg d^−1^	10^5^ conidia ml^−1^	Branches 100 µm^−1^
Δ*crn-1*	3.1±0.2	28.0±4	0.6	1.5±0.1
WT	13.5±0.6	43.5±5	1.5	0.3±0.03

(mean ± standard error).

### Changes in actin cytoskeleton in Δ*crn-1* mutant

**Figure 5:**
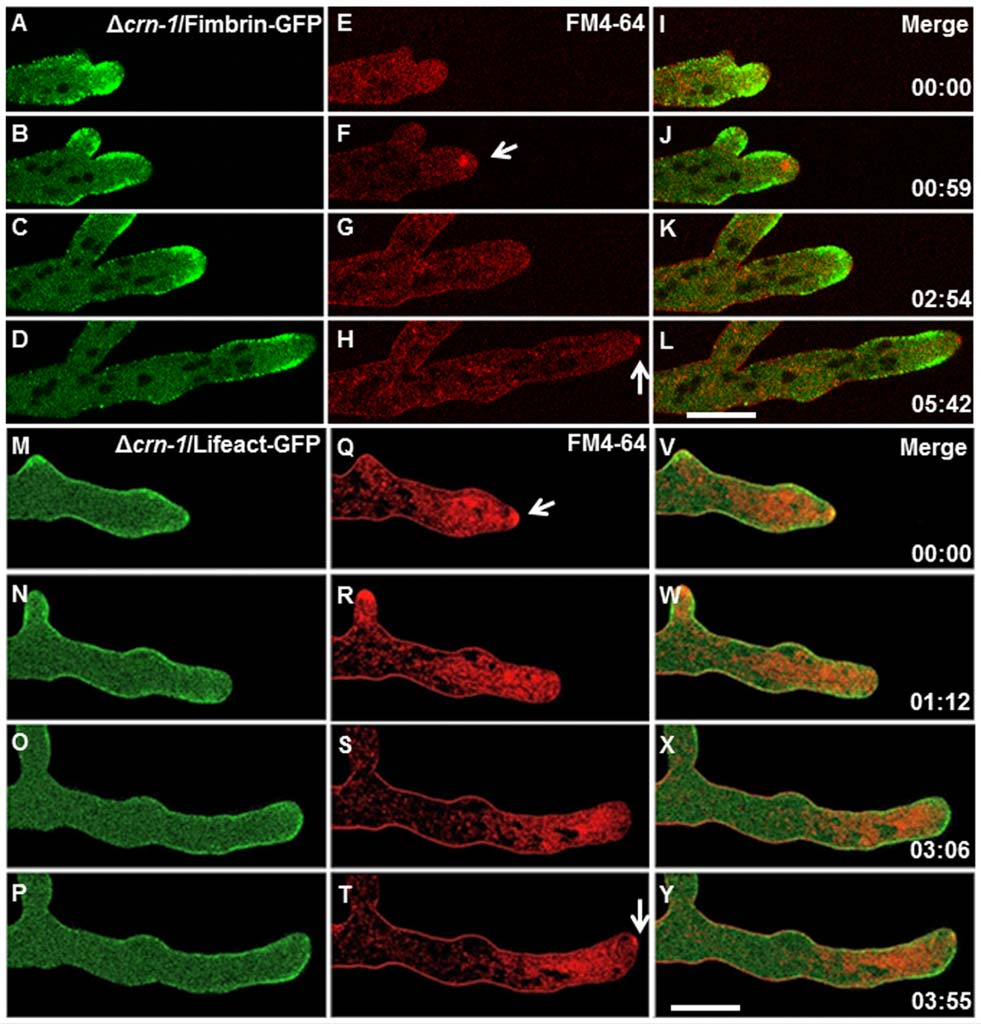
Dynamics of the subapical endocytic collar and Spk behavior in the Δ*crn-1* mutant. (A–L) Subapical endocytic collar of fimbrin, (A–D) Fimbrin-GFP, (E–H) FM4-64 staining and (I–L) merge of Fimbrin-GFP and FM4-64. (M–Y) actin distribution shown with (M–P) Lifeact-GFP, (R–T) staining with FM4-64 and (V–Y) merge of Lifeact-GFP and FM4-64. White arrows point to the presence of the Spk. Time in min:sec. Scale bar = 10 µm. Note. The hypha Q–T was exposed to FM4-64 for a longer time prior to the start of the sequence, hence the stronger red signal.

The location of fluorescently labeled actin and fimbrin was examined in the Δ*crn-1* mutant strain (Fig. 5). Fimbrin localized to patches along the hyphal cortex, with a conspicuous accumulation in a subapical collar, immediately subtending the area occupied by the Spk (Fig. 5A, 5B, 5D). Notably, when tip polarity was transiently lost and primarily isotropic expansion occurred, the subapical collar of fimbrin patches relocated into the apical dome (Fig. 5C). Coincidentally, the Spk retracted into the subapical region and disappeared (Supplementary Movie S3).

As Delgado-Alvarez *et al.* [11] previously reported for the WT strain of *N. crassa*, we also detected a strong signal for F-actin in the Spk core, and in the patches of the subapical endocytic collar of the Δ*crn-1* mutant expressing Lifeact-GFP. However, the distribution and dynamics of actin in the Δ*crn-1* mutant changed continuously during the observed growth periods. These changes correlated with changes in the Spk and in the morphology of the growing tip. Periodically, the strong Lifeact signal of F-actin in the apex disappeared and simultaneously the FM4-64 stained Spk dispersed (Fig. 5N and Fig. S1). As long as a Spk and its actin core were present, constant growth ensued and the morphology of the growing tip became decidedly hyphoid (Fig. 5P). When the Spk disintegrated, growth seemed to slow down and the tip became hemispherical (Fig. 5M–P). Another visible change accompanying the disappearance of the Spk was the relocation of F-actin patches from the subapical collar towards the tip, invading the area previously occupied by the Spk (Fig. 5N–5O; Supplementary Movie S4).

### Δ*crn-1 mutant* and endocytosis

**Figure 6:**
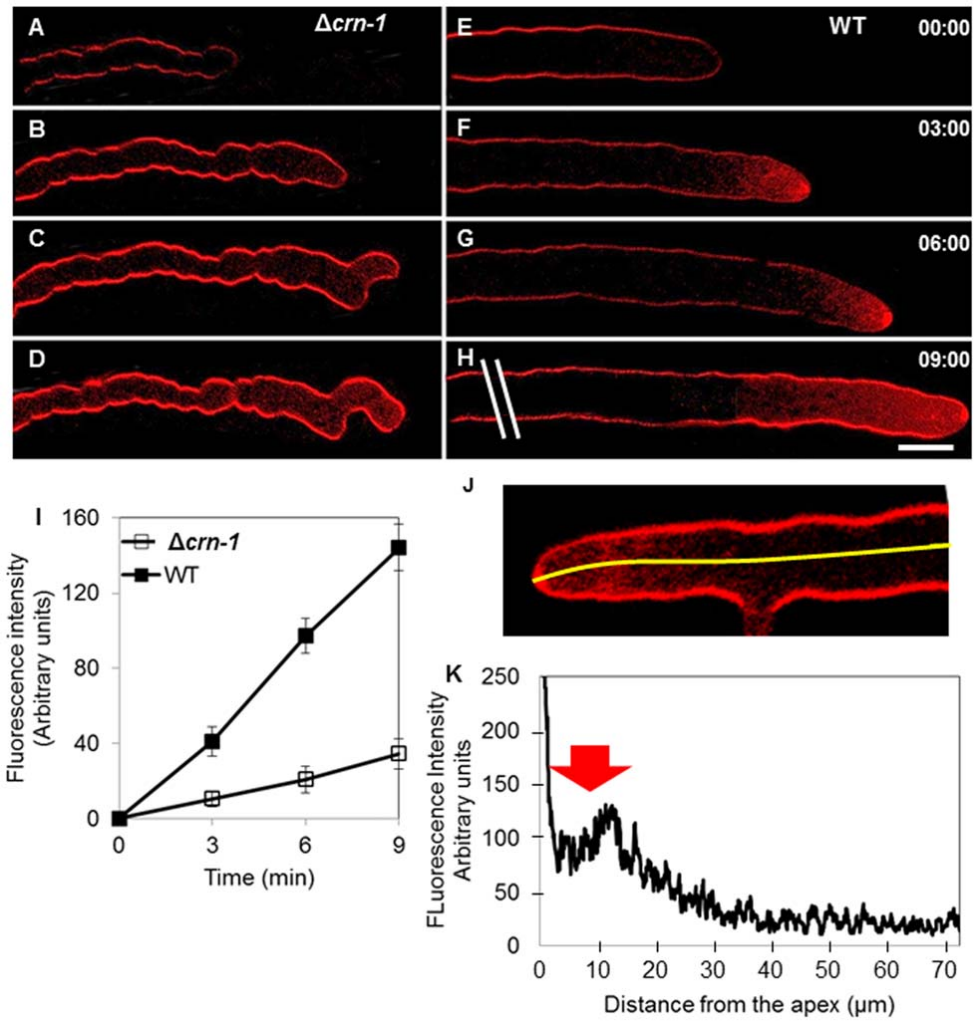
Comparative rates of internalization of the endocytic marker FM4-64. (A–D) Δ*crn-1* mutant and (E–H) WT (I) Graph of fluorescence intensity in a subapical cytoplasmic region (10 µm from the tip; the area measured averaged 50 pixels) in the Δ*crn-1* mutant (n = 30) and the WT (n = 30). (K) Graph of fluorescence intensity along the first ∼70 µm (yellow line) from the apex of the WT hypha shown in J (n = 10). Arrow points to the endocytosis region. Time in min:sec. Scale bar = 10 µm.

The rate of internalization of the endocytic marker FM4-64 was markedly reduced in the Δ*crn-1* mutant (Fig. 6). Upon addition of the dye, the plasma membrane of the WT and mutant became labeled immediately (Fig. 6A, 6E). After three minutes, however, the fluorescence intensity in the cytoplasm of the WT strain was 3-times higher than in Δ*crn-1* mutant. The difference in cytoplasmic fluorescence intensity between WT and mutant persisted during the observation period (Fig. 6I). The average time for full staining of the Spk with FM4-64 was ∼7 min in Δ*crn-1* mutant but only ∼2 min in WT (n = 30). A fluorescence profile along the hyphal tube showed maximum intensity coinciding with the position of the subapical endocytic collar (Fig. 6J, 6K).

### Spk behavior in Δ*crn-1* mutant

**Figure 7:**
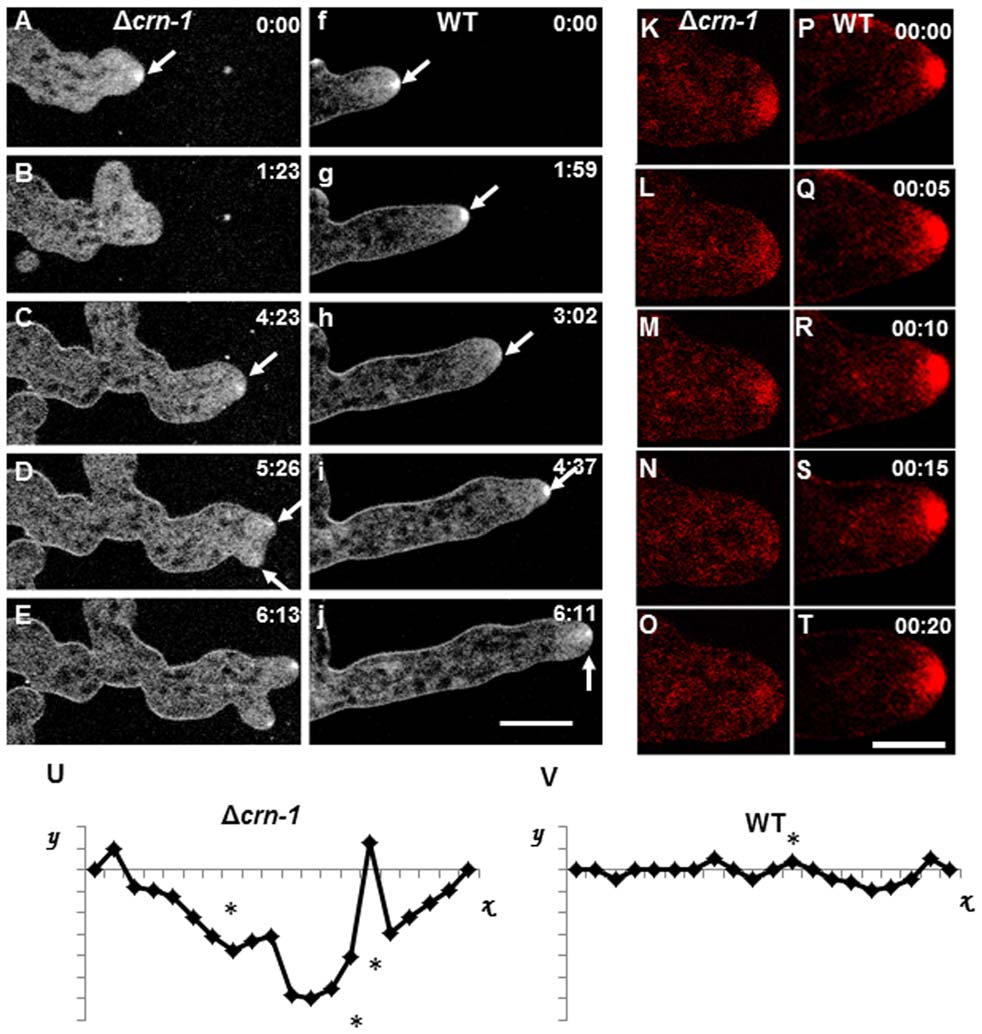
Comparative morphogenesis and Spk behavior revealed by staining hyphae with FM4-64. (A–E) Δ*crn-1* mutant and (F–J) WT strain. Arrows show the Spk. Details of Spk dynamics in (K–O) Δ*crn-1* mutant and (P–S) WT strain. Spk trajectories plotted relative to the growth axis (abscissa). Time in min:sec. Scale bar = 10 µm.

FM4-64 staining showed a smaller Spk in coronin deficient hyphae (4.6±0.4 µm^2^; n = 30) and with ovoid shape rather than the larger spherical body of WT (8.9±0.6 µm^2^; n = 30) (Fig. 7). The Spk of Δ*crn-1* mutant was unstable, i.e. its integrity and presence at the apex was only sustained for short periods (Fig. 7). Spk disassembly and reassembly led to frequent changes in shape and growth directionality. When the Spk became disrupted, the hyphal tip tended to grow in an isotropic fashion and lost directionality (Fig. 7A–7E). Notably, in the absence of coronin, time lapse sequences (Supplementary Movies S5 and S6) showed alternating periods of polarized and non-polarized growth producing small and large shape changes of the hyphae that were often accompanied by a loss in growth directionality. Sometimes, the FM4-64 stained Spk appeared to split into two smaller Spks each giving rise to an apical branch with a well-defined Spk (Fig. 7C–E; supplementary Movies S5). Often, incipient branches formed but aborted coinciding with the disassembly of the Spk. In addition to intermittent appearance and disappearance, the Spk of the mutant showed a much more erratic trajectory than the WT Spk (Fig. 7U–7V).

The hyphal elongation rate of the Δ*crn-1* mutant was only 23% of that of the WT strain (p<0.05), however, the wider diameter and meandering morphology of the mutant hyphae makes this comparison misleading (Table 1). Biomass production was a more reliable parameter to compare growth. Accordingly, the Δ*crn-1* mutant retained 64% (p<0.05) of the growth capacity of the WT strain.

### Conidiogenesis and conidium germination in the Δ*crn-1* mutant

**Figure 8:**
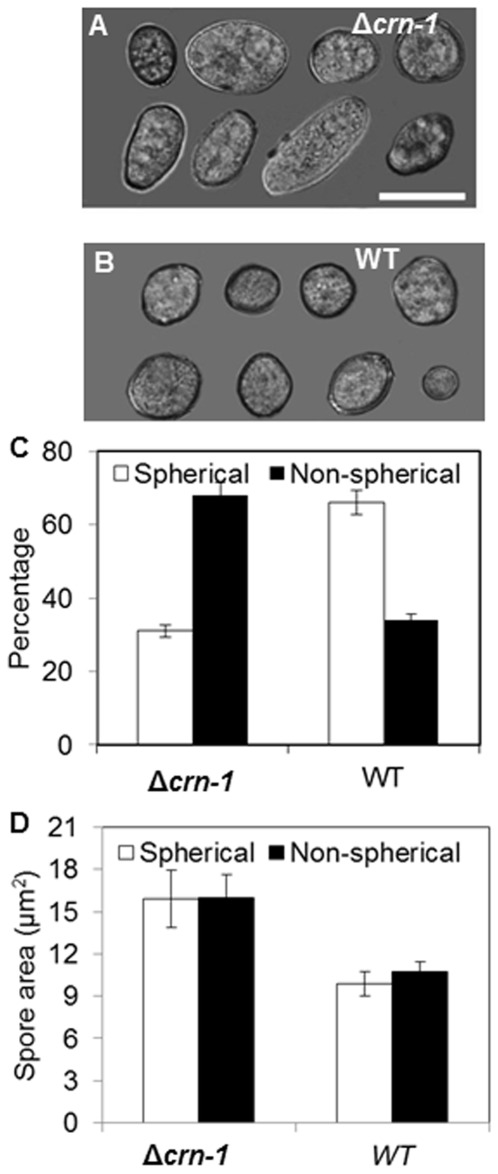
Comparison of conidial morphology and size. Conidiophores of (A) Δ*crn-1* mutant and (B) WT strain. Composite image of conidia representing the most common shapes in (C) Δ*crn-1* mutant and (D) WT strain. (E) Relative abundance of spherical and non-spherical conidia in the Δ*crn-1* mutant and WT strain. (F) Average size of conidia in the Δ*crn-1* mutant and WT strain. The error bars represent the 95% confidence interval. Scale bar = 5 µm.

Conidium size and shape were markedly affected by the loss of coronin. More than 60% of mutant conidia had non-spherical shapes (Fig. 8E), contrary to the WT that had spherical or near-spherical conidia in the same proportion as non-spherical conidia in the mutant (Fig. 8D, 8E). On average, conidia of the Δ*crn-1* mutant were twice as big as WT conidia, independent of their shape (Fig. 8F). A 2-day colony of Δ*crn-1* mutant produced less than the half of the conidia (0.6×10^5^ conidia ml^−1^) formed by the WT strain in the same period (1.5×10^5^ conidia ml^−1^).

**Figure 9:**
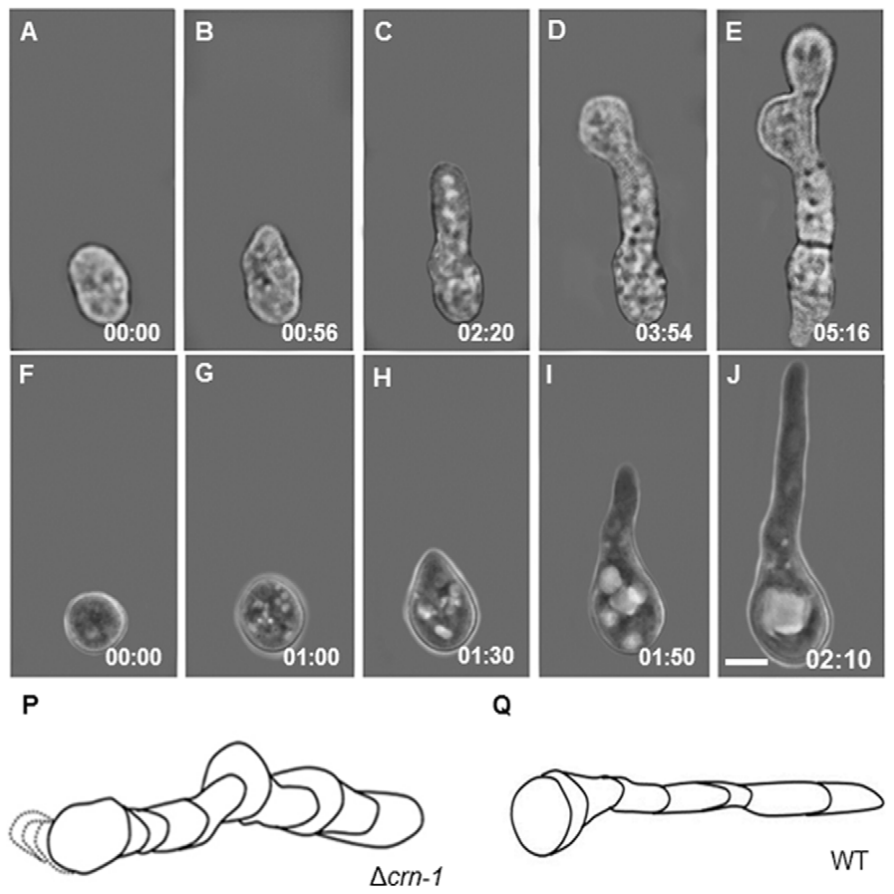
Conidial germination. (A–E) Time series of the Δ*crn-1* mutant by bright field microscopy and (F–J) WT strain by phase contrast microcopy. Reconstruction of the morphological differences during conidial germination of above sequences over longer time periods (K) Δ*crn-1* mutant and (L) WT. Time in h:min. Scale bar = 5 µm.

Conidial germination was significantly different in the Δ*crn-1* mutant (Fig. 9). The rate was much slower, the emerging germ tube wider and was more prone to meandering than that in the WT. Often, the elongation of the germ tube was interrupted by budding-like processes that yielded one or more buds in linear succession until a tube developed. The final appearance was that of a septated germ tube (Fig. 9E).

**Figure 10:**
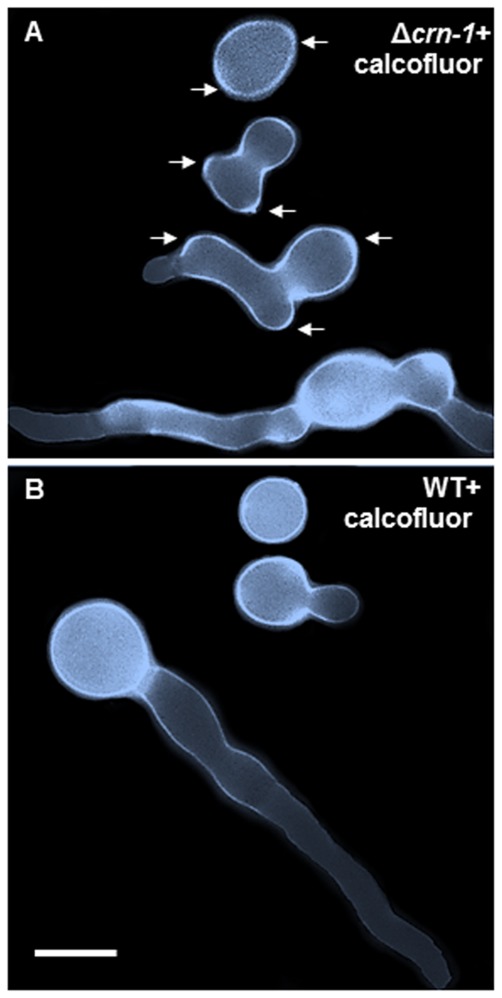
Cell wall differences revealed by calcofluor white. Composite image of cells stained with calcofluor white (0.01%) and arranged to show different developmental stages in (A) the Δ*crn-1* mutant and (B) the WT strain. Arrows point to some of the uneven distribution of cell wall accumulation. Scale bar = 5 µm.

The irregular staining pattern of the cell walls of conidia and their germ tubes with calcofluor white indicated that misdirected synthesis and/or excessive deposition of cell wall material accompanied the aberrant morphology of the coronin null mutant conidia and germlings (Fig. 10).

## Discussion

### Coronin an ABP component of the subapical endocytic collar

The existence of a specialized region of the actin cytoskeleton in the subapex of fungal hyphae was first discovered in *Aspergillus nidulans* and characterized by the presence of patches of certain actin-binding proteins (ABPs) namely AbpA, AmpA, SlaB [14] and fimbrin [13] forming an annular arrangement or “collar” at a short distance from the hyphal tip. Indirect evidence was presented correlating this collar with the major site of endocytosis in growing hyphae. In hyphae of *N. crassa*, Delgado-Alvarez *et al.* [11] detected a similar subapical collar of fimbrin and also found that another ABP, Arp2/3 complex, was part of this subapical collar. By using Lifeact to visualize actin, the relationship between the collar of ABP patches and the entire actin cytoskeleton of a hypha of *N. crassa* became clear [11], [32]. Coronin can now be include as another component of the subapical collar in *N. crassa* and hence another likely gear in the endocytosis machinery of this fungus. The colocalization of coronin with other ABPs in the same patches supports the notion of an integrated function of all these ABPs in endocytosis [29]–[31], [33].

The disruption of actin cables, when hyphae were treated with anti-actin polymerization reagents, caused the disassembly of the Spk with its associated actin skeleton, and the subsequent migration of the collar cortical patches into the cell apex. The greater resistance of actin patches to depolymerization, compared to filamentous actin, can be attributed to the stabilizing presence of different types of ABPs in the patch [11], [25].

### Morphogenetic consequences of coronin deletion

The morphology and behavior of the Δ*crn-1* mutant gave us useful insight into the role of the cytoskeleton in polarized growth, i.e. hyphal morphogenesis. Remarkably, despite the absence of coronin, the fungus remained functional both in being capable of growing and undergoing hyphal morphogenesis and conidiogenesis although both functions were visibly impaired. Overall growth was reduced by 36%; hyphal morphology and directionality were deeply affected as polarized growth was turned on and off intermittently. The hyphal profiles were unevenly undulated or crenulated and the cell wall showed a markedly irregular thickness. This host of alterations could be ascribed to intermittent disturbances of the pattern of exocytic vesicle migration that has been predicted generate a normal hypha with a regular hyphoid shape [1], [3]. Seemingly, in the absence of coronin, the actin cytoskeleton becomes somewhat unstable impacting intermittently the assembly of the Spk and thus the orderly process of cell wall construction. Presumably, the actin-rich core of the Spk is the focal target of the cytoskeletal disturbance. In the absence of a Spk polarized exocytosis becomes disorganized producing erratic delivery of cell wall building components and thus irregular wall thickness and altered hyphal morphology. Although, the lack of coronin strongly affects the cell, it is not an essential factor for hyphal growth, as other components of the endocytic machinery i.e. Sla B, that has shown as essential in *A. nidulans* [34].

The finding that CRN-1-GFP was absent in the apex of the WT strain seems surprising since deletion of *crn-1* caused adverse effects on apical activities (Spk and exocytosis). Therefore the disturbances in Spk behavior and apical morphogenesis observed in the Δ*crn-1* mutant must be indirect effects and thus evidence that coronin impacts the function of the actin cytoskeleton, i.e., the subapical and apical actin cytoskeletons are functionally interrelated, It remains to be determined to what extent any reduction in endocytosis may have also affected Spk behavior. Altogether our findings indicate that a defective actin cytoskeleton can support polarized hyphal growth albeit with sometime serious distortions; evidently, normal or optimum hyphal morphogenesis requires an intact actin cytoskeleton.

### Coronin is required for Spk stability and dominance

The Spk is believed to function as a supply center of secretory vesicles needed for polarized expansion of the cell wall and plasma membrane at the hyphal tip. The advancing Spk generates an orderly gradient of cell wall construction responsible for the characteristic (hyphoid) shape of hyphae and the directionality of their growth [35]–[37]. As shown recently, the Spk of *N. crassa* harbors in a stratified manner the microvesicles responsible for chitin synthesis (chitosomes) and the macrovesicles involved in ß-1,3-glucan synthesis [38]. First Girbardt [39] and later Bartnicki-García *et al.* [36], Riquelme *et al* [37] and most convincingly Bracker *et al.* [40] correlated the position and trajectory of the Spk with growth directionality.

**Figure 11:**
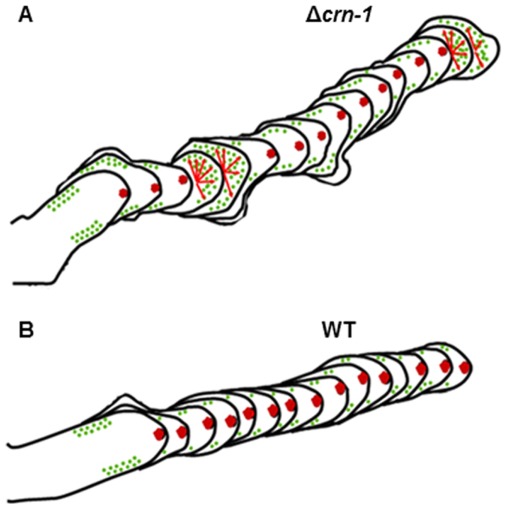
Reconstructions of morphogenetic sequences from time lapse movies of the Δ*crn-1* mutant and the WT strain; the mutant shows a lack of directionality and temporal loss of polarized growth, the green dots show the places of endocytic patches and how they are misplaced in the mutant when the Spk is not present, the small arrows show the shifts between polarized growth and isotropic growth.

The variable and somewhat erratic morphology of the coronin null mutant allowed for the observation of the relationship between actin and Spk assembly in dynamic detail, and to assess its morphogenetic consequences. When a well-defined Spk was present, there was a strong actin signal in the Spk core. The hyphae grew rapidly and generated tubes with distinct hyphoid shapes. When the Spk was absent, the actin core dispersed, polarity was diminished or lost and cell expansion became isotropic and the tips adopted a hemispheroid shape. Most of the morphological changes observed may be correlated with repeated failure to maintain a fully functional Spk core. This alternation in Spk integrity produces the convoluted/crenulated morphology of the hyphae in the Δ*crn-1* mutant. As shown vividly in Supplemental Movie S3, the recurrent losses of Spk integrity coincided with the dispersal of the fimbrin-labeled collar patches from their subapical location thus suggesting that both disturbances were caused by a generalized failure of the coronin-deprived actin cytoskeleton (Fig. 11).

The undulated profile of the Δ*crn-1* mutant hyphae plus the irregular thickness of the cell wall are strong indicators that the orderly migration of wall-building vesicles was altered intermittently by the absence of the stabilizing effect of coronin. The bumps in the crenulated hyphal profile may result from 1) an irregularity of the integration and disintegration of the Spk thus creating alternating periods of hyphoid and isotropic growth, respectively; 2) formation of spurious secondary growth centers in the proximal subapex, some of which appear to be abortive branching attempts that were unable to maintain polarized growth. Clearly, in the absence of coronin the Spk has difficulty maintaining its integrity and forward movement, losing intermittently its ability to coordinate the flow of exocytic vesicles. As a result, growth turns isotropic and also secondary ephemeral growth centers may appear in the immediate subapical region. Coronin seems to have important stabilizing function maintaining the organization of the entire apical growth apparatus and the subapical endocytic collar.

### Are endocytosis and exocytosis linked?

The spatial proximity and functional complementarity between delivery of secretory vesicles in the apical dome and recovery of plasma membrane and protein in the sub-apical collar poses intriguing questions of possible cross regulation between the two processes in fungal hyphae [41]–[42]. The role of exocytosis on endocytosis appears straightforward, with endocytosis being the consequence of excess accumulation of plasma membrane discharged by exocytosis. However, the reverse is less clear but is being actively explored [41]–[42].

Calculations of membrane deposited by apical exocytosis and the amount needed to extend the plasma membrane indicate that an excess of membrane is usually produced during hyphal elongation (Bartnicki-Garcia unpublished). According to this assumption, a primary role for endocytosis would be to maintain a correct membrane balance in the growing hyphae. Together with membrane recycling, endocytosis may also serve to recover proteins integrated into the membrane thus creating a tandem relationship between exocytosis and endocytosis. Our observations on the incorporation of FM4-64 indicate that the absence of coronin reduced but did not eliminate endocytosis. Therefore, as argued above, it would seem that coronin while not essential for the operation of the subapical (endocytic) actin-collar, it does assure an optimum rate of endocytosis.

### Two functionally different actin cytoskeletons in hyphal growth

Our finding that coronin plus fimbrin and Arp2/3 are present in a subapical collar, but not in the apical dome region of hyphae of *N. crassa* supports the notion that two functionally different actin-cytoskeletons are involved in the polarized growth of a hypha, a subapical collar made actin and ABPs patches and actin cables. While the actin cytoskeleton in the hyphal apex drives exocytosis, the one in the subapex would be involved in driving endocytosis. Presumably, one reason for the involvement of several ABPs in the subapex but not in the apex of a hypha is the distinct requirements to perform intrusion vs. extrusion of vesicles in and out of the cytoplasm, respectively. These two processes face totally different obstacles; foremost, endocytosis must overcome the enormous turgor of the hyphal cytoplasm, whereas the final step of exocytosis would be greatly facilitated by cytoplasmic turgor.

Since we have confirmed that coronin was indeed deleted from the Δ*crn-1* mutant; coronin, while not essential, does play an important role keeping the actin cytoskeleton and perhaps the entire cytoskeleton operating normally. The latter conclusion would be in concordance with the long known fact that coronin has a role linking microtubules to F-actin [43]–[44]. The incomplete but remarkable resilience of the Δ*crn-1* mutant is probably due to functional compensation conferred by other elements of the cytoskeleton. Apparently, the degree of redundancy varies in other organisms as evident by the fact that coronin deletion in yeast causes no obvious changes in the phenotype and hence seems fully compensated [43].

The exact mode of operation of coronin is not yet known. Findings on other organisms suggest that coronin operates in conjunction with other ABPs, notably cofilin and Arp2/3, to promote both actin assembly and disassembly [45]. Coronin is the switch between activating and inhibiting the Arp2/3 complex, controlling the its recruitment to filaments or blocking binding sites for the complex, to finally affects the actin turnover in patches [46]. Altogether our findings indicate that a defective actin cytoskeleton can support polarized hyphal growth albeit with sometime serious distortions; evidently, normal or optimum hyphal morphogenesis requires an intact actin cytoskeleton.

## Materials and Methods

### Strains and culture conditions

Strains used in this study are listed in Table 2. Strains were maintained on Vogel's minimal medium (VMM) supplemented with 2% sucrose. Cultivation procedures were according to standard techniques [47].

**Table 2:** Materials used. *N. crassa* strains, plasmids and oligonucleotides.

Name	Genotype, description, or sequence	Reference
**Strains**		
FGSC2225	*mat A Wildtype*	FGSC
FGSC9717	*mat A-Δmus^−51^-his-3^−^*	FGSC
FGSC0202.4	*mat a Δcrn-1*	FGSC
TRM100-RE01	*mat A Δcrn-1; his-3^−^*	This study
TRM101-RE02	*mat A Δcrn-1; his-3^+^::*P*ccg-1-Lifeact-egfp^+^*	This study
TRM102-RE03	*mat A Δcrn-1; his-3^+^::*P*ccg-1-fim-1-sgfp^+^*	This study
TRM103-RE04	*mat A Δcrn-1 his-3^+^::*P*ccg-1-bml-1-sgfp^+^*	This study
TRM24-OC17	*mat A crn-1-sgfp- his-3^+^*	This study
TRM25-OC18	*mat A crn-1-mCherry^+^*	This study
TRM104-RE05	*mat A crn-1-mCherry^+^; his-3^+^::*P*ccg-1-Lifeact-egfp^+^*	This study
TRM105-RE06	*mat A crn-1-mCherry^+^; his-3^+^::*P*ccg-1-Lifeact-egfp^+^*	This study
TRM106-RE07	*mat A crn-1-mCherry^+^; mat A his-3^+^::*P*ccg-1-fim-1-sgfp^+^*	This study
**Plasmids**		
pMF272	P*ccg-1*-s*gfp^+^*	[52]
pRM-24-OC17	P*ccg-1-crn-1-sgfp^+^*	This study
pRM-25-OC18	P*ccg-1-crn-1-mCherry^+^*	This study
pRM-49-OC30	P*ccg-1*-lifeact-e*gfp^+^*	[11]
pRM-08-D02	P*ccg-1-fim-sgfp^+^*	[11]
pLS-NG01	P*arp2*-*arp-2-sgfp^+^*	[11]
pMF309	P*ccg-1-bml-1*-*sgfp^+^*	[52]
**Oligonucleotides**		
COR-XBAI F	*5′ GC* ***TCTAGA*** *ATGCGCCGAAGCCAAGCC* * 3′*	
COR-PACI R	*5′ CC* ***TTAATTAA*** *CGACCTAGCAGCCTCGAGC* * 3′*	

Restriction enzymes sequence in bold.

### Construction of coronin-fluorescent proteins-containing plasmids

Standard PCR and cloning procedures [48] were used to fuse the *sgfp* gene to the carboxyl terminus of *crn-1*. The *crn-1* gene was amplified by PCR from *N. crassa* (FGSC 2489) genomic DNA. Primers used are also listed in Table 2. PCR was performed in an Apollo Thermal Cycler with Platinum Hi-Fi Taq polymerase (Invitrogen, Carlsbald, CA) according to the manufacturer's instructions. The amplified and gel-purified PCR product were digested with *XbaI* and *PacI* and ligated into *XbaI*- and *PacI*-digested plasmid pMF272 (GenBank accession no. AY598428) for GFP tagging and pJV15-2 for mChFP [38]. The resulting expression plasmids pRM24-OC17 and PRM25-OC18, respectively, were verified by sequencing at Eton Biosciences (San Diego, CA). The expression in both vectors is under control of the *ccg-1* promoter [49]–[50]

### Transformation protocols, transformant selection and crosses

Electroporation was used to transform conidia of *N. crassa* Δ*mus-51::his-3* strain (FGSC9717) with non-linearized plasmids (Table 2) using a Bio-Rad Gene Pulser and standard settings (capacitance, 25 µF; 1.5 kV; resistance, 600 Ω) as previously described [51]. Prototrophic his+ transformants were screened for the expression of GFP or mChFP by epifluorescence microscopy as described before [52]. Selected heterokaryotic transformants were back-crossed to a WT mat a strain (FGSC2489), using synthetic crossing medium (SCM) supplemented with 1% sucrose and 2% agar [47], and fluorescent progeny from isolated ascospores were stored for further studies.

In order to target expression vectors to the *his*-3 locus in a Δ*crn-1* mutant background we produced a Δ*crn-1/his-3^−^* double mutant by crossing with Δ*mus-51::his-3^−^* strain (FGSC9717). Progeny colonies that grew on 0.3 mg ml^−1^ hygromicyn selection media but not on in medium without histidine were selected. The double mutant Δ*crn-1/his-3^−^* was transformed following the procedure described above, in order to express fimbrin-GFP and Lifeact-GFP.

This double mutant Δ*crn-1/his-3^−^* ^1^ was also used to test the complementation of the deletion mutant, together with the functionality of the Crn-1-GFP fusion. We transformed the double mutant with plasmid pRM24-OC17, and the WT phenotype and growth rate were recovered as well as the distribution of fluorescence.

### Double labeling: coronin-mChFP plus other ABPs-GFP

To observe the relationship between coronin, actin and other ABPs regulators of F-actin, namely the Arp2/3 complex and fimbrin and actin, we generated heterokaryons through vegetative fusion of strains, expressing CRN-1-mChFP and fimbrin-GFP, ARP-2-GFP and Lifeact-GFP. For this, a VMM plate was inoculated with spores of both strains, and incubated for 10 h at 28°C. Subsequently, colonies were screened for hyphae expressing both fluorescent makers using laser scanning confocal microscopy.

### Growth kinetics, branching and conidiation rates

To phenotypically characterize the Δ*crn-1* mutant, we measured its colony extension rate, hyphal elongation rate, biomass production, lateral branching frequency and conidiation rates, and characterized the colonial and hyphal morphology during growth and germination as described below. All experiments were performed as triplicates and in comparison to a wild type control.

#### Growth rate and biomass production

Ten µl of conidial suspension (1.5×10^5^ spores ml^−1^) were inoculated on the edge of 15 cm diameter VMM plates and incubated at 28°C for 48 h. The mean colony extension rate (cm d^−1^) was calculated after measuring the mycelium diameter every 6 h until the plates were filled. The hyphal elongation rate was measured in time-lapse movies recorded by phase contrast with an inverted Axiovert 200 microscope (Carl Zeiss, Gottingen, Germany) using a 100× (PH3)/1.3 N.A. oil immersion objective. Images were captured at 4 s intervals for 5 min, and analyzed with the Axiovision Rel. 4.6.3 software. The mean elongation rate was calculated from the frame-to-frame differences, and data was stored and processed in Excel® (Microsoft, Redmond, WA).

For biomass production measurements, 10 µl of conidial suspensions (1.5×10^5^ spores ml^−1^) were inoculated onto VMM plates overlaid with previously dehydrated and weighed dialysis membrane and subsequently incubated at 28°C for 24 h. The developed mycelium was lifted off the agar with the membrane, dried and weighed with an analytical balance (Sartorius, Bradford, MA). Biomass was calculated as the weight difference between the dialysis membranes before and after incubation, and expressed in mg d^−1^.

#### Branching frequency

Strains were inoculated on VMM plates and incubated at 28°C for 24 h, then observed on an Olympus SZXILLB2-100 (Olympus, Tokio, Japan) stereomicroscope at a magnification of 400×. Images were captured with an Olympus DP70 CCD camera and analyzed with the accompanying software. The number of lateral branches of 30 leading hyphae was counted in the first 100 µm from the tip (branches/100 µm).

#### Conidiation rate

To measure conidia production, VMM plates were inoculated with the WT and Δ*crn-1* mutant strains and incubated at 28°C for several days, i.e. until sufficient conidiophores were developed. Five ml of 1 M sorbitol were used to rinse off the conidia from the culture and collected. Spore concentration in the suspension was determined using a Neubauer cell counting chamber (American Optical, Buffalo, NY).

### Membrane and cell wall fluorescent staining

Using the “inverted agar block method” [53], GFP-expressing strains were incubated with 5 µM FM4-64 (Molecular Probes, Eugene, OR) to stain the plasma membrane and organelle membranes [16]. The cell wall was stained with 0.01% calcofluor white (American Cyamamid Co. Brook, NJ).

### Laser scanning confocal microscopy

Fluorescence and phase contrast microscopy of the coronin null mutant and WT strains was performed on an inverted laser scanning microscope (LSM-510 Meta, Carl Zeiss, Göttingen, Germany) equipped with an argon ion laser for excitation at 488 nm for GFP and a He-Ne laser for excitation at 543 nm for mChFP and with filters to capture the emission signal between 515–530 nm for GFP and 590–700 for mChFP. A 100× (PH3)/1.3 N.A. oil immersion objective was used, and laser intensity was kept to a minimum to reduce photobleaching and phototoxic effects. Time-lapse imaging was performed at scan intervals of 0.5 to 4.5 s for periods up to 40 min. Images were recorded with 512×512 pixels and 300 dpi resolution using the implemented LSM-510 software (version 3.2; Carl Zeiss), and evaluated and converted into. AVI movie files with the associated LSM 510 Image Examiner program. Fluorescence images were simultaneously captured with phase contrast images using one photomultiplier tube to detect the transmitted light from the laser illumination [9]. Image processing for the preparation of figures was performed with Adobe Photoshop CS3 Extended (Adobe Systems Inc, San Jose, CA).

### Electron microscopy

For transmission electron microscopy germlings were grown on a thin, sterile, deionized dialysis membrane overlying VMM at 23°C. The cells were cryofixed by plunging them rapidly into liquid propane cooled to −186°C with liquid nitrogen. Cryofixed cells were freeze substituted in acetone containing 2% osmium tetroxide and 0.05% uranyl acetate at −85°C for 48 hrs. After the completion of freeze substitution, cells were slowly warmed to room temperature by first transferring them to −20°C for 2 h, then to 4°C for 2 h and finally to room temperature for 1 h. After being rinsed in 100% acetone, the cells were infiltrated with epoxy resin, flat-embedded between a Teflon-coated glass slide and Aclar film, and polymerized at 60°C for 24 h. After resin polymerization, cells were thin-sectioned on a Leica ultramicrotome (Leica Microsystems Inc., Bannockburn, IL) and post stained for 10 min in 2% uranyl acetate in 50% ethanol and for 5 min in lead citrate. Sections were examined using on an FEI CM12S TEM (FEI Electronics Instruments, Co., Mahwah, NJ) at 100 kV coupled to a Gatan 689 CCD digital camera (1024×1024 pixel area; Gatan Inc., Pleasanton, CA). For all imaging methods, final figures were constructed using Adobe Photoshop 7.0 (Adobe Systems Inc, San Jose, CA).

## Supporting Information

Figure S1 Spk and actin behavior in the Δ*crn-1*. (A–E) Actin labeled by Life-act-GFP marker present in the core of the Spk and the sub apical collar. (F–J) Spk stained with FM4-64. The Spk and its actin skeleton are assembled and disassembled at the same time. When both are not present, the actin patches from the subapical collar migrate to the apical dome. The white arrows show the presence of the Spk and its actin skeleton. Time in min:sec.
(TIF)

Movie S1 Movie shows growth of normal hyphae stained with FM4-64 and the protein Coronin labeled with sGFP.
(AVI)

Movie S2 Movie shows growth of normal hyphae stained with FM4-64 and the protein Coronin labeled with sGFP during an event when the Spk disassemble and the coronin patches move towards the tip.
(AVI)

Movie S3 Movie shows labeling of fimbrin::sGFP in a Δcrn-1 background. Fimbrin patches relocate into the apical dome and the Spk fells back into the subapical region and dissolves.
(AVI)

Movie S4 Movie shows labeling of actin with life-act and FM4-64 in a Δcrn-1 background. Actin is present at the Spk and in the subapical collar. During disappearance of the Spk stained with FM4-64, the Lifeact-stained F-actin core of the Spk also entirely disassembles, and cortical F-actin patches relocates from the subapical collar towards the tip, invading the area previously occupied by the Spk.
(AVI)

Movie S5 Movie shows Spk behavior in a Δcrn-1 backgorund stained with FM4-64. The Spk disassembles and reassembles leading to frequent changes in shape and growth directions. An event of apical branching is also observed.
(AVI)

Movie S6 Movie shows Spk behavior in a WT strain stained with FM4-64.
(AVI)
