# Supplementary figures and images for: Coronin Is a Component of the Endocytic Collar of Hyphae of Neurospora crassa and Is Necessary for Normal Growth and Morphogenesis
Source: PLoS One. 2012 May 31;7(5):e38237. doi: 10.1371/journal.pone.0038237 (PMC3365027; doi:10.1371/journal.pone.0038237)

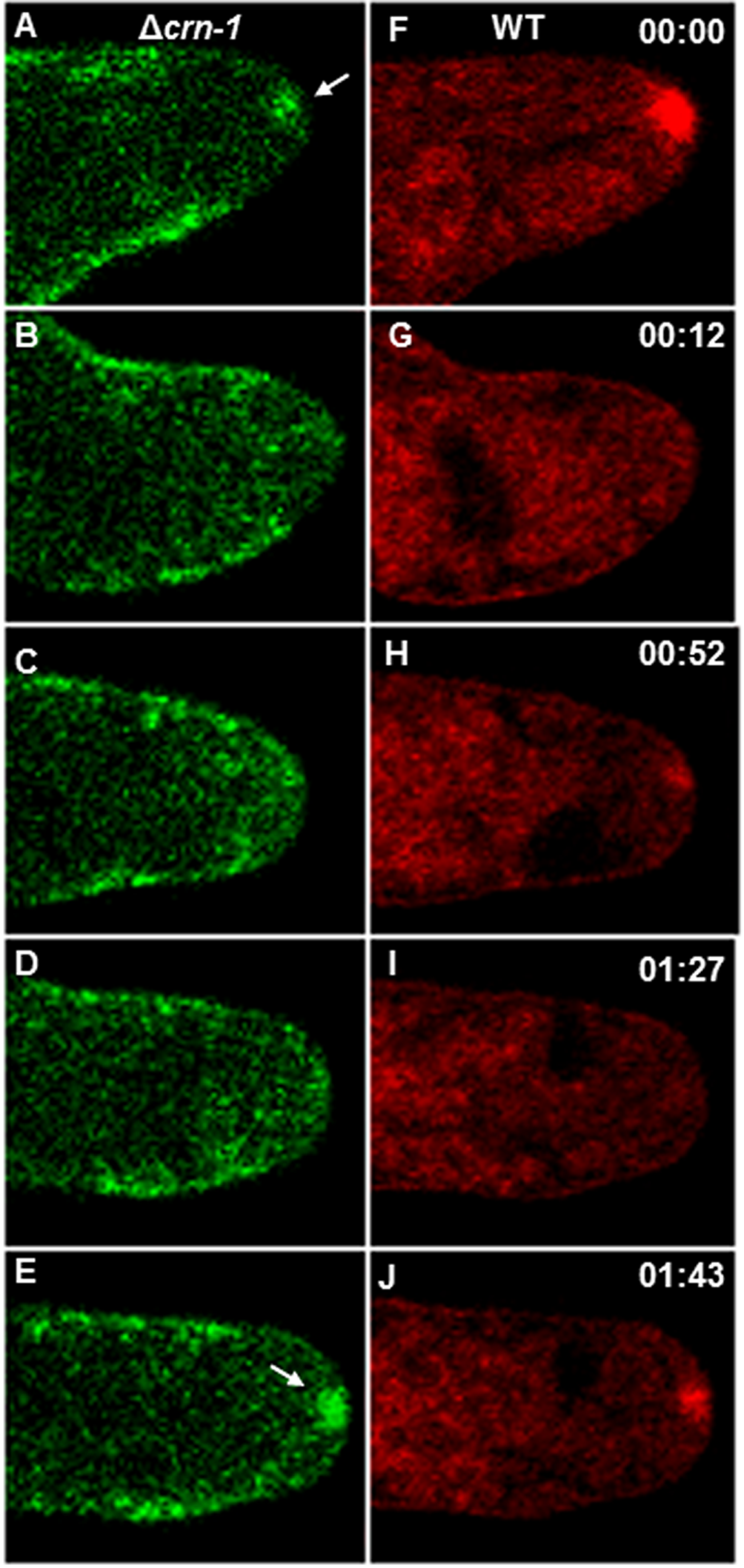

Supplement: Figure S1 — Spk and actin behavior in the Δcrn-1. (A–E) Actin labeled by Life-act-GFP marker present in the core of the Spk and the sub apical collar. (F–J) Spk stained with FM4-64. The Spk and its actin skeleton are assembled and disassembled at the same time. When both are not present, the actin patches from the subapical collar migrate to the apical dome. The white arrows show the presence of the Spk and its actin skeleton. Time in min:sec. (TIF) [file pone.0038237.s001.tif]
